# Case of Rheumatism Producing Phlegmon

**Published:** 1805-08-01

**Authors:** 


### Case of Rheumatism producing Phlegmon.

It is rather taken for granted, than established as a truism, that rheumatism is a specific species of inflammation. There are, however, I apprehend, very sufficient grounds for such a distinction. Nosologists have constantly given it a place among the phlegmasiæ. Depending, like all the other species, for its existence upon the continued action of the proximate cause, it has, in truth, a common claim to such arrangement. But while it exhibits various phenomena in common with acute symptomatic inflammatory fever, as it is called, and that other more chronic form of inflammatory excitement, which is termed hectic fever, it has other properties, which are so many distinguishing marks that are sufficiently obvious, and need not be particularised here minutely. It is the characteristic termination of rheumatism, when seated in a theca or capsular ligament, to throw out serum, together with a greater or less proportion of the coagulable lymph of the blood, and of the proper secreted fluid of the diseased part, forming a matter of some consistence, very different from common inflammatory effusion. The more ordinary seat of rheumatism is in the ligamentous membranes investing both the tendons and the extremities of the bones; it affects also the muscles of the body, the investing membranes as well of the bones generally as of the cavities of the body, if not the viscera themselves. In all these cases, a similar effusion probably occurs in the interstices between the constituent fibres of the part. Suppuration is as rare a concomitant of rheumatic as of arthritic inflammation; and I apprehend that either the one or the other of the latter runs into, or rather is converted into the suppurative inflammation, only when (exceeding its common boundary) it is extended, in a violent degree, to the neighbouring cellular cellular substance, the common connecting medium of the body, the proper seat of that species of inflammation. That rheumatism is seated in the subordinate arteriæ anastomicæ is, I believe, the general opinion of pathologists, and appears to have more than probability on its side. That the lymphatics, if not the nerves, may likewise be involved in the derangement, no one probably will deny, however he be disposed to refuse his assent to the hypothesis of Dr. Latham upon this point.

That property of certain diseases, which renders them convertible into other diseases of the same class, does not, perhaps, throw any particular light on their essence or specific character in the present state of medicine: yet, as it forms a part of their natural history, it derives hence some importance in a practical view, and should, therefore, be carefully noticed by him who wishes conscientiously to discharge the office of conducting diseases through their natural processes. The conversion of erysipelas into the phlegmonoid inflammation, and vice versa, is an occurrence sufficiently familiar. Erysipelas having attained a certain point, is sometimes overtaken, as it were, by the phlegmonoid inflammation, and the two diseased actions maintain, with some irregularity, their proper characteristics at one and the same time, in one and the same member. But it happens much more frequently in such mixtures, that one of the two morbid actions takes the lead, the other ceasing. A similar conversion with respect to phlegmonous and rheumatic inflammation is a much more rare occurrence. Dr. Darwin talks of the suppurative rheumatism, in his chimerical work, entitled Zoonomia; and other instances of such a concurrence may be found in medical authors. The following is so striking a case of such an hybrid disease, that I submit it to the public eye.

Thomas Ains, aged 16, a thin spare lad, of sanguine habit, after exposure to wet and cold, was attacked, October 10, 1804, with severe pain in one knee, and pyrexia. In about forty-eight hours the knee and thigh had swoln so enormously as to be nearly double its former bulk. At this period violent pain, quickly succeeded by swelling, attacked the other knee; then both ancles, and successively the arms, wrists and fingers. In a few days an abscess was produced on one of the fore fingers, which, on being punctured, discharged genuine pus freely. Now another abscess (preceded by much local pain in the clavicle and shoulder) nearly as large as a hen's egg, arose just above the left clavicle, which, on being punctured,

 discharged discharged about two ounces of genuine pus also. It may be remarked, that the latter tumour being seated immediately over the subclavian vessels, had a strong pulsatory motion evident to the eye across the chamber. Not a drop of blood escaped on evacuating it; but at the expiration of about three hours afterwards, eight or ten ounces of blood escaped from the cavity of the abscess, probably derived from ulceration of a small branch of the carotid artery. The hæmorrhage was quickly restrained by the spontaneous contraction of the vessel. The breathing was laborious for several days, accompanied with constant and smart diarrhœa; pulse hard, ranging from 120 to 130. Topical and general blood-letting, relaxants with blisters to the chest, fomentation and poultices, were the principal measures adopted for the relief of the patient, but with so little efficacy that he died on the twelfth day of the disease, completely exhausted, the dyspnœa increasing.

*May* 6, 1805.

